# Reminiscences of the Insane

**Published:** 1887-04-02

**Authors:** 


### Reminiscences of the Insane.

By a Mental Physician.

(*Continued from page 373.*)

Poem on Madness.

We have already referred to the poetical compositions of the insane. Many years ago an insane gentleman with whom, while we held office in ——— Asylum, we had many pleasant games of chess, wrote a large number of cantos on "Madness," from which not a few flowing lines could be cited, as, for example, the following:—

"Oh!—vain all self-sought cure of mental ill!—
For while on earth sin's rampant powers shall reign,
Weak reason's stream, and intellect's proud will
Distorted aye shall be, and maniac's pain
Be thine—oh man!—Yes! Madness yet will gain—
(So dares the muse in faith to prophesy):—
A more extended sway o'er Earth's domain;—
And till the soul's deep fount we purify,
That growing ill the body's healing will defy."

In this poem the author describes with considerable ability some of his fellow-patients in the institution. He hears beneath his window—

"The plaintive airs of female song, Ophelia's madness brings
To memory's ears, and through the pain'd soul rings
The knell of reason!"

He recognises the sorrowful results of insane inheritance in the lines—

"God's wrath hath told
That some ancestral parent in the vale
Of by-gone years hath sinn'd; and these their sins bewail."

The poet-patient thus apostrophises another patient:

"Oh, I have often watched thy rapid walk,
Thou maniac mother of a beauteous throng
Of offspring fair,—and marked the frequent talk
With other minds; yes, I have heard thy song,
In Italy, or Spain, or France's tongue,
Sweetly commingling with the harp's soft tones;
And oft thy friendly smiles and accents 'mong,
Have passed a lively hour; but the sad moans
Of husband and of child all happiness dethrones."

It would occupy too much of the limited space at our command to continue this poetical picture-gallery, however interesting the portraiture to those who knew the patients. It may be added that he speaks of the depths of unhappiness; he describes as

"Where ne'er the lyre before
Hath strung its plaining chords to man's worst woe,
And told of human grief the hidden store."

On this line the poet notes that, "Surely no physical or mental suffering can be comparable to the loss of reason; therefore is it that the author thus designates it as man's worst woe."

Soothing Influence of Composition

The author refers to "the soothing effect which the mere composition of verse has over the mind," and he knows not how it would have fared with him had he not possessed this resource; for in the four months of his *alleged* insanity (say rather excess of sensibility than privation of intellect) he composed, in addition to the present poem, some eighteen or twenty fugitive pieces, several of which exceed a hundred, and one has nearly five hundred lines. Alas, but for this unlooked-for resuscitation of his long-neglected harp, and for the power, such as he possesses, of handling its sympathetic chords, the author's fearful probation of confinement would have been more trying.

Good Effects of Exercise.

Everyone knows the salutary effects of regular exercise in the treatment of those insane persons for whom it is appropriate. The author of the poem states in a note the course which he pursued during one of his asylum experiences. He occupied the first hours of the morning in original composition; took ample exercise for a couple of hours before dinner; accompanied those patients who were in a condition to go out to the walled gardens; and then, having measured the distance round the principal path, about 120 yards, he was in the practice of marking on a slate every round, whether in walking or running, and in this he often accomplished by measurement eight or even nine miles in the two hours; and so much did he practise his lungs by running, that after commencing with five or six times round the garden, without any other stoppage than scoring the marks, he gradually reached up to 20, 30, 50, and once to 80 times, or about five miles and a half on a stretch. He observes that when so running he has often and often recalled the facts asserted by South American travellers, that there are runners who attend on journeys, and keep up with a trotting horse, for fifteen and twenty miles without stopping.

Circular Insanity.

There is a form of insanity which has received the name of "Circular," in which the patient passes through several distinct and opposite phases of disorder, returning again (unless he recover) to the point of the vicious circle from which he started—the unhappy state prefigured by the fabled wheel of Ixion. Of this the author of *Madness* was a striking example, and he himself records, that "after a period of the most fearful and sinful depression, he suddenly arises out of it, and becomes altogether a totally changed being. From silence and sorrow, the soul emerges to freedom and irrepressible joy; and from a state of intellectual weakness bordering almost upon imbecility, to one of unwonted energy and fluency."

Patient in Chains.

Before this patient was admitted into the asylum where we resided, he had experienced the old system of treatment in an institution to which Lord Shaftesbury frequently referred in conversations and speeches, as among the worst of the Metropolitan Asylums. He says: "The hand that now writes,—with its fellow-hand,—and even the feet, have been chained for weeks and days together in solitary confinement; and occasionally, also, by a longer chain to a staple in the floor in the common day-room, with some twenty or thirty other patients. So also at night have the metal handcuffs been used." Here he got worse and worse, more impatient and excited, and his friends then concluded to remove him. In less than a fortnight after his removal to ——— Asylum he greatly improved, although for some time there was mental depression. He describes the change in the following lines of his poem, in which he says that it was impossible that

"The mind
Of Herculean strength, could, thus confin'd,
Re-breathe, uninjur'd, liberty's sweet air;
The vigorous frame, to ruffian hands assign'd,
In vain could aid the intellect's right care,
And bad to worse he came, while lingering sadly there.

"What else could spring from chains and dungeons dire?
(For not alone with crime do these unite)
This anger'd form, in retrospection's ire,
E'en now recalls the peace-destroying sprite
Which roved his fancy through, and deadening quite
All reason's power—left the demented one
In worse than ignorance's Stygian night.

"All vain the effort to constrain by force,

The impetuous mind, or stay its fierce career;

And conscious then that 'twere a vain resource,

'Mid anger's fitful tempests, thus to steer,

Some Guardian Angel (ah! to memory dear!)

In mercy pointed to where Ouse's stream

With gentle current winds that terrace near,

On which Sol's earliest and resplendent beam

Shines o'er York's vale with peaceful ray and rest supreme."

Before passing away from this patient and his poem, it may be interesting to record that Southey wrote a kindly letter to him, in which he expressed a fear that the subject he had chosen (Madness) was of too exciting a nature for him, adding that five-and-thirty years before he had borrowed from an old Spaniard for his motto the words *In labore quies.* "Long ago," he continued, "I was warned by experience never to proceed continuously with any work which I had in hand after I had begun to *dream* of it; and this is the reason why I have always several works in progress." He advises him to feed his ear by perusing those poets who have written best in stanzas,—Fairfax's *Tasso*, Phineas Fletcher's *Purple Island*, his brother Giles Fletcher, all that Daniel and Drayton have written in the octave stanzas. If I have not mentioned Spenser, it is not from forgetfulness of a poet whom I look to more than any other as my master, but, because while, in all other respects, he is one of the greatest (and to me the most delightful) of all poets, his language is peculiarly his own. Poetry is as much an art as architecture; and if you would practise it, you must study poets as your brother studied cathedrals."

Letter from Southey.

(*To be continued.*)

